# The Perioperative Revolution in Muscle-Invasive Bladder Cancer - Progress, Caution, and the Latin American Perspective

**DOI:** 10.1590/S1677-5538.IBJU.2026.0158

**Published:** 2026-03-18

**Authors:** Caio Vinícius Suartz, Paulo Roberto Salustiano de Carvalho, Ricardo de Albuquerque Freitas, Augusto Modesto de Souza, Daher Cezar Chade, Leopoldo Alves Ribeiro, José Henrique Dallacqua Santiago, Daniel de Freitas G. Soares, Karin Marise Jaeger Anzolch, Roni Fernandes de Carvalho, Fernando Korkes

**Affiliations:** 1 Sociedade Brasileira de Urologia Disciplina de Câncer de Bexiga Rio de Janeiro RJ Brasil Disciplina de Câncer de Bexiga, Sociedade Brasileira de Urologia - SBU, Rio de Janeiro, RJ, Brasil; 2 Northern Ontario School of Medicine Department of Urology Thunder Bay Ontario Canada Department of Urology, Northern Ontario School of Medicine, Thunder Bay, Ontario, Canada; 3 Universitário Pedro Ernesto Serviço de Urologia Rio de Janeiro RJ Brasil Serviço de Urologia, Hospital Universitário Pedro Ernesto, Rio de Janeiro, RJ, Brasil; 4 Hospital do Centro Oncológico Aristides Maltez Serviço de Urologia Salvador BA Brasil Serviço de Urologia, Hospital do Centro Oncológico Aristides Maltez, Salvador, BA, Brasil; 5 Universidade de São Paulo Instituto do Câncer de São Paulo, Serviço de Urologia São Paulo SP Brasil Serviço de Urologia, Instituto do Câncer de São Paulo, Universidade de São Paulo - USP, São Paulo, SP, Brasil; 6 Faculdade de Medicina do ABC Departamento de Urologia Santo André SP Brasil Departamento de Urologia, Faculdade de Medicina do ABC, Santo André, SP, Brasil; 7 Santa Casa de Porto Alegre Departamento de Urologia Porto Alegre RS Brasil Departamento de Urologia, Santa Casa de Porto Alegre, Porto Alegre, RS, Brasil; 8 Hospital Moinhos de Vento Departamento de Urologia Porto Alegre RS Brasil Departamento de Urologia do Hospital Moinhos de Vento, Porto Alegre, RS, Brasil; 9 Faculdade de Ciências Médicas da Santa Casa de Misericórdia de São Paulo Departamento de Urologia São Paulo Brasil Departamento de Urologia, Faculdade de Ciências Médicas da Santa Casa de Misericórdia de São Paulo, São Paulo, Brasil

## COMMENT

The therapeutic landscape of bladder cancer has evolved at an unprecedented pace over the past decade. From refinements in the management of non–muscle-invasive disease to transformative advances in muscle-invasive bladder cancer (MIBC), the field has shifted from a historically radical surgical paradigm to a biologically driven, multimodal approach (1). Radical cystectomy, once the unquestioned standard, is now integrated into perioperative systemic strategies that include neoadjuvant chemotherapy, perioperative chemo-immunotherapy, perioperative immunotherapy with antibody-drug conjugates, and, in selected cases, trimodal therapy, as endorsed by contemporary NCCN guidelines (2).

The NIAGARA trial marked a pivotal milestone in cisplatin-eligible patients with MIBC. The incorporation of perioperative durvalumab with gemcitabine–cisplatin demonstrated pathological complete response (pT0N0M0) rates of 37.3% and a 2-year overall survival of 82.2%, outcomes not previously achieved with chemotherapy alone in randomized settings. These results led to rapid guideline incorporation of perioperative chemoimmunotherapy and redefined expectations for curative-intent treatment (2, 3).

More recently, at the American Society of Clinical Oncology Genitourinary Cancers conference, the results of EV-304/KEYNOTE-B15 were presented, challenging a two-decade paradigm in muscle-invasive bladder cancer. For years, cisplatin-based neoadjuvant chemotherapy represented the cornerstone of systemic management in eligible patients. In this phase 3 trial, the combination of enfortumab vedotin plus pembrolizumab was evaluated in cisplatin-eligible patients and demonstrated a pathological complete response rate of 55.8%, compared with 32.5% with standard chemotherapy. Two-year overall survival was 86.9% versus 81.3%, respectively (hazard ratio, 0.65; p=0.0029), establishing a new benchmark for perioperative systemic therapy.

In parallel, KEYNOTE-905 (EV-303), recently published in the New England Journal of Medicine, extended this therapeutic advance to a historically underserved population: patients ineligible for cisplatin. In a high-risk cohort in which approximately 80% presented with stage III disease, perioperative pembrolizumab plus enfortumab vedotin achieved a pathological complete response rate of 57.1% and an estimated 2-year overall survival of 79.7%, with a hazard ratio for event-free survival of 0.50. These findings challenge the long-standing paradigm in which cisplatin-ineligible patients were directed to upfront cystectomy without systemic intensification.

Together, these studies redefine the perioperative landscape of muscle-invasive bladder cancer and signal a shift from chemotherapy-centered strategies to biologically intensified combinations capable of delivering unprecedented pathological and survival outcomes.

However, these advances warrant critical contextualization, particularly in Latin America. The region reports an incidence of approximately 4–5 cases per 100,000 inhabitants per year and a mortality rate of approximately 2.1 per 100,000, according to GLOBOCAN estimates (4). Despite this epidemiologic burden, Latin American populations remain underrepresented in landmark perioperative trials. In NIAGARA, only 9.6% of patients were enrolled from South America. In KEYNOTE-905, racial representation was similarly limited, with only 1.2% Black participants and 2.4% reporting multiple racial backgrounds (3,5).

This underrepresentation becomes more pronounced in subgroup analyses. In KEYNOTE-905, the magnitude of benefit appeared more pronounced among White participants, whereas confidence intervals for event-free survival (HR 0.29; 95% CI 0.24–1.02) and overall survival (HR 0.76; 95% CI 0.31–1.82) crossed unity in other racial groups. A similar phenomenon was observed in NIAGARA within South American subsets (EFS HR 0.55; 95% CI 0.29–1.03). While these analyses are exploratory and underpowered, they underscore the need for broader representativeness before universal extrapolation (5).

A second critical consideration is financial toxicity. Bladder cancer is among the malignancies associated with the highest lifetime treatment costs (6). The integration of perioperative immunotherapy and antibody–drug conjugates further expand cumulative systemic exposure and intensifies resource utilization across the continuum of care. In NIAGARA, patients received four cycles of neoadjuvant therapy followed by eight cycles of adjuvant durvalumab. In KEYNOTE-905, three cycles of neoadjuvant pembrolizumab plus enfortumab vedotin were followed by up to one year of combination therapy. These strategies extend treatment duration well beyond conventional cisplatin-based neoadjuvant paradigms, with direct implications for drug acquisition costs, infusion capacity, monitoring requirements, and management of immune-related toxic effects.

The economic impact is particularly relevant in resource-constrained health systems. In Brazil, between 2019 and 2023, only one judicial claim within the public health system involved enfortumab vedotin for metastatic urothelial carcinoma; between 2024 and 2025, approximately 36 such claims were filed, representing a 36-fold increase. This surge reflects litigation-driven access to high-cost therapies and signals mounting fiscal pressure on both public and private payers. Such patterns illustrate how rapid therapeutic innovation, when not accompanied by structured incorporation policies, may shift decision-making from evidence-based allocation to judicial mandates.

Despite therapeutic advances, substantial disparities in access to contemporary oncologic care persist in Brazil, a country marked by pronounced geographic and socioeconomic heterogeneity. Limited availability of high-complexity treatments within the public system contributes to delays in diagnosis and definitive management, reinforcing inequities in oncologic outcomes. In this context, adoption of intensified perioperative regimens should be accompanied by rigorous pharmacoeconomic analyses, formal budget-impact modeling, and region-specific implementation strategies. Ensuring sustainability while preserving equitable access will be central to translating these advances into meaningful population-level benefit (7, 8).

A lastly notable finding is that the EV-304/KEYNOTE-B15 population included a higher proportion of cT3–T4 tumors than in earlier perioperative chemotherapy trials. Patients with more advanced local disease and a higher risk of occult micrometastases may derive greater absolute benefit from systemic intensification. This difference in case mix provides important context for interpreting the magnitude of benefit without detracting from the trial's internal validity.

The next frontier must be biomarker-driven selection. Identifying molecular predictors of response could maximize oncologic benefit while limiting unnecessary exposure to immune-related and antibody–drug conjugate–associated toxicity, particularly given that adverse events were reported in 100% of patients treated with enfortumab vedotin plus pembrolizumab. Precision strategies may also support bladder-sparing approaches in carefully selected patients. However, longer follow-up remains essential: beyond early pathological and survival signals, durability of benefit, late toxicity, quality of life, and functional outcomes must define the true value of intensified perioperative therapy. Ultimately, success will be measured not only by hazard ratios, but by durable, patient-centered benefit.

The trajectory of bladder cancer management is undeniably optimistic. The magnitude of clinical gain is unquestionable. The field is advancing at unprecedented speed and with unprecedented biological sophistication. Yet progress must be accompanied by equity.

Innovation must not only change guidelines; it must change outcomes across populations..

## References

[1] Reis LO (2026). Bladder cancer immunotherapy in 2025: ALBAN, CREST, POTOMAC, and the winner is BCG!. Int Braz J Urol.

[2] Flaig TW, Spiess PE, Abern M, Agarwal N, Bangs R, Buyyounouski MK (2024). NCCN Guidelines® Insights: Bladder Cancer, Version 3.2024. J Natl Compr Canc Netw.

[3] Powles T, Catto JWF, Galsky MD, Al-Ahmadie H, Meeks JJ, Nishiyama H (2024). Perioperative durvalumab with neoadjuvant chemotherapy in operable bladder cancer. N Engl J Med.

[4] Bray F, Laversanne M, Sung H, Ferlay J, Siegel RL, Soerjomataram I (2024). Global cancer statistics 2022: GLOBOCAN estimates of incidence and mortality worldwide for 36 cancers in 185 countries. CA Cancer J Clin.

[5] Vulsteke C, Adra N, Danchaivijitr P, Sabadash M, Rodriguez-Vida A, Zhang Z (2026). Perioperative enfortumab vedotin and pembrolizumab in bladder cancer. N Engl J Med.

[6] Scilipoti P, Moschini M, Li R, Lerner SP, Black PC, Necchi A (2025). The financial burden of localized and metastatic bladder cancer. Eur Urol.

[7] Sistema e-NatJus. Conselho Nacional de Justiça Fórum da Saúde [Internet].

[8] Korkes F, Silveira MA, Tocci F, Pedrotti C, Teich VD, Camargo-de-Barros LH (2024). Lawsuits against the Brazilian Unified Health System regarding bladder/ureteral cancer. Braz J Oncol.

